# Jellyfish Collagen in the Mediterranean Spotlight: Transforming Challenges into Opportunities

**DOI:** 10.3390/md23050200

**Published:** 2025-05-03

**Authors:** Ainara Ballesteros, Raquel Torres, Maria Pascual-Torner, Francisco Revert-Ros, Jose Tena-Medialdea, José Rafael García-March, Josep Lloret, Josep-Maria Gili

**Affiliations:** 1 Institute of Environment and Marine Science Research, Universidad Católica de Valencia (IMEDMAR-UCV), C. Guillem de Castro, 94, 46001 Valencia, Spain; raquel.tcardenal@ucv.es (R.T.); josetena@ucv.es (J.T.-M.); jr.garcia@ucv.es (J.R.G.-M.); 2 Doctoral School, Universidad Católica de Valencia, 46001 Valencia, Spain; 3 Department of Marine Biology and Oceanography, Institute of Marine Sciences (ICM-CSIC), Passeig Marítim de la Barceloneta 37-49, 08003 Barcelona, Spain; mpascual@icm.csic.es (M.P.-T.); gili@icm.csic.es (J.-M.G.); 4 Mitochondrial and Molecular Medicine Research Group, Facultad de Medicina y Ciencias de la Salud, Universidad Católica de Valencia San Vicente Mártir, 46001 Valencia, Spain; fj.revert@ucv.es

**Keywords:** aquaculture, biomaterial, blue biotechnology, by-catch, fishing, industrial application

## Abstract

Research increasingly highlights jellyfish as a sustainable alternative to other animal species, particularly for its collagen, which has versatile applications in blue biotechnology. This review explores the properties of jellyfish-derived collagen, extraction techniques, and its diverse industrial applications based on the current scientific literature. With a particular focus on research in the Mediterranean Sea, we underscore the role of the order Rhizostomeae as jellyfish species with high collagen content and provide an overview of the main sources for jellyfish harvesting, including active fishing, by-catch, and aquaculture. In the Mediterranean basin, the blooming species *Rhizostoma pulmo*, *Cotylorhiza tuberculata*, and *Rhopilema nomadica* represent a valuable opportunity to harness their nutraceutical benefits, as well as their potential for the development of biomaterials in tissue engineering and regenerative medicine. Although jellyfish fishing is not yet well-established in the region, ongoing collaborative projects with fishermen’s guilds are focused on promoting circular and blue economy strategies to valorize jellyfish as an innovative resource. Additionally, jellyfish aquaculture emerges as a promising alternative for ensuring a sustainable supply, with the Rhizostomeae *Cassiopea* spp. demonstrating significant potential for biotechnological applications.

## 1. Introduction

Jellyfish often form large aggregations, commonly referred to as jellyfish blooms or outbreaks [1,2]. Increasing evidence suggests that jellyfish biomass may be rising across numerous coastal marine ecosystems worldwide, including the Mediterranean Sea, Black Sea, East Bering Sea, and Yellow Sea, among others [3,4], largely as a result of intensifying human-induced pressures [1,5]. However, the lack of comprehensive data spanning broad temporal and spatial scales hinders the ability to draw a reliable conclusion from a global perspective [4,6]. Several anthropogenic factors have been identified as key drivers of jellyfish blooms. Climate change, for instance, has been shown to enhance asexual reproduction rates in response to rising seawater temperatures [7,8]. Additionally, species translocation via maritime activities, such as ballast water discharge and hull biofouling, facilitates the introduction and spread of non-indigenous species (NIS) [9]. Habitat modification through coastal urbanization (i.e., ocean sprawl) has also contributed to jellyfish population increases by providing artificial substrates that support planulae settlement and polyp proliferation [10]. Furthermore, overfishing has led to the depletion of jellyfish competitors and predators, disrupting trophic interactions and potentially promoting jellyfish dominance in certain ecosystems [1].

Jellyfish are often described in negative terms, portrayed as a nuisance or “pest” species due to their interference with human activities [11,12]. Their blooms have detrimental effects on marine aquaculture, resulting in high fish mortality and the spread of disease, as well as negatively impacting the fishing industry by clogging and damaging nets [13]. Moreover, jellyfish outbreaks can disrupt industrial infrastructure, such as power plants and desalination facilities, by obstructing cooling-water intake systems [14]. However, the most significant socio-economic impact is observed in the recreational use of coastal areas, where jellyfish stings directly affect tourists seeking swimming conditions during the peak season [15].

Despite these socio-economic impacts, jellyfish are increasingly recognized as contributors to various ecosystem services [11]. They represent an essential component of marine food webs, serving as a food source for commercial fish species such as *Boops boops* [16] and for the leatherback sea turtle, *Dermochelys coriacea* [17]. Moreover, jellyfish fulfill important ecological roles by acting as floating substrates, shelter, and habitat for a variety of marine organisms in open-ocean ecosystems, where physical refugia are scarce [11]. Under eutrophic conditions, jellyfish have been linked to the maintenance of water quality through their top-down control of the food web, thereby supporting ecosystem stability [18].

As terrestrial habitats continue to face overexploitation, marine organisms have gained attention as promising sources of bioactive compounds with valuable biotechnological applications, including collagen-based products [19,20]. Currently, most commercial collagen products are derived from mammalian sources, primarily bovine and porcine. However, these sources are under increasing scrutiny due to environmental concerns and restrictions associated with religious beliefs [19,21]. Marine-derived collagens are predominantly obtained from vertebrate species such as fish, fish by-products, and other aquatic organisms [19]. Recently, invertebrate species, particularly jellyfish, have emerged as viable alternative sources [19,20,21]. Jellyfish-derived collagen exhibits high biocompatibility, a low risk of allergenic responses, and minimal potential for zoonotic disease transmission, positioning it as a safer and more sustainable alternative to traditional mammalian collagens [21,22]. Furthermore, as invertebrates, jellyfish offer a unique source of collagen with distinct functional and physicochemical properties, making them especially suitable for a wide range of applications [19,20].

In this context, this review aims to promote, summarize, and discuss current research on jellyfish collagen in the Mediterranean Sea, demonstrating its broad versatility and potential as an alternative collagen source, as well as its exploitation sources. To the best of our knowledge, this is the first review to focus in detail on the Mediterranean Sea for the valorization of jellyfish as an innovative resource.

## 2. Jellyfish Collagen

**Figure 1:**
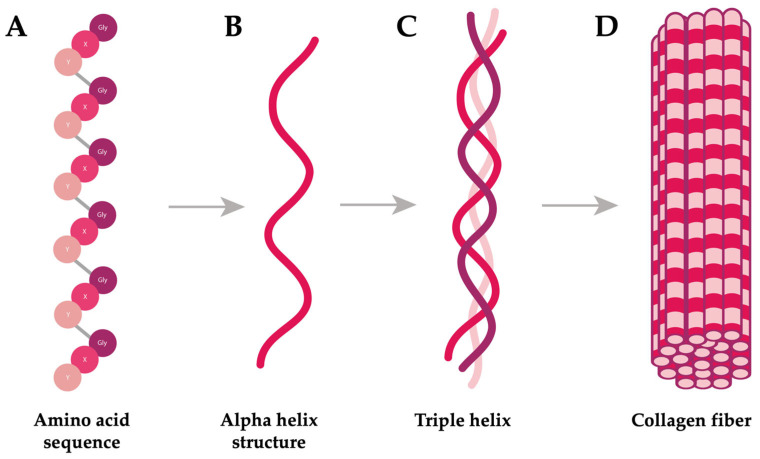
Structural diagram of collagen including (**A**) primary structure, (**B**) secondary structure, (**C**) tertiary structure, and (**D**) quaternary structure. Amino acid sequence: Gly stands for glycine, and X and Y represent two other amino acids, usually proline and hydroxyproline.

Collagen is the most abundant structural protein in animals and a key component of the extracellular matrix (ECM). Its name in Greek means “gum” (*kolla*) “producer” (*gen*), highlighting its connective role in biological structures [23]. The primary structure of collagen consists of repeating triplets of glycine (Gly), proline (Pro), and hydroxyproline (Hyp), which are fundamental to its stability and function (Figure 1A). These polypeptide chains are folded into an alpha helix type, a characteristic secondary structure of collagen (Figure 1B). Three of these helices then intertwine to form a right-handed triple helix, constituting the tertiary structure (Figure 1C). Multiple triple helices subsequently aggregate in a staggered arrangement to form collagen fibrils, the fundamental quaternary structural units responsible for the mechanical strength and integrity of connective tissues (Figure 1D) [23,24,25].

Among the more than 20 types of collagen, classified based on their structural features and amino acid composition, the most abundant are Types I, II, III, IV, and V. Type I collagen is the most prevalent and extensively studied form, accounting for over 90% of the organic matrix of bone and serving as the primary collagen in tendons, skin, ligaments, and most connective tissues in vertebrates. It typically adopts a triple helix structure consisting of two α1 chains and one α2 chain, which is critical for its structural integrity and tensile strength [23,26].

Jellyfish collagen primarily consists of Types I and II, with some occurrences of Types III, IV, and V, and the same species may contain different collagen types [21]. However, this classification is based on vertebrate collagen standards, even though invertebrate collagen has structural differences. Because of this, some authors argue that jellyfish collagen should not follow the conventional mammalian classification and propose a unique category called “Type 0” [27]. Proteomic analyses confirm its different peptide sequences, reinforcing the idea that it is a unique protein [27]. Jellyfish collagens have fewer amino acid residues and a lower melting temperature compared to mammalian type I fibrillar collagen [22,28,29]. This is consistent with the fact that invertebrate collagen chains typically have more glycine and fewer proline or hydroxyproline residues than mammalian collagens [22,29]. Additionally, jellyfish collagen presents differences in binding motifs, such as a lack of α2β1 integrin-binding motifs, which reduces cell adhesion in some cell types, while not affecting others whose adhesion relies on alternative β1 integrins and possibly on heparan sulfate interactions [27]. Moreover, jellyfish collagen deviates due to negligible calcified tissues and a high collagen-to-insoluble extracts ratio [27].

In jellyfish, the ECM, known as the mesoglea, is located between the epidermis and endodermis and is responsible for their gelatinous consistency. Mesoglea, and, therefore, collagen, play essential roles in maintaining body structure by serving as a substrate for cell attachment and migration [30], regulating buoyancy [31], and enabling locomotion in aquatic environments [32]. Additionally, the gelatinous nature of jellyfish allows for an increased body volume, enhancing feeding efficiency under low food concentrations [33]. Collagen also participates in critical biological processes of jellyfish, such as wound healing, regeneration, and morphogenesis [34,35].

Human cells recognize jellyfish collagen and exhibit comparable cytotoxicity, cell adhesion, and proliferation rates to those observed with mammalian type I collagen [22]. However, the mechanism of cell adhesion differs, with heparan sulfate proteoglycans playing a more prominent role [27]. Finally, jellyfish collagen stimulates the immune system without triggering IgE-mediated allergic reactions [36].

## 3. Jellyfish Rich in Collagen That Inhabit the Mediterranean Sea

### 3.1. Order Rhizostomeae

Scyphomedusae (Cnidaria: Scyphozoa), also known as true or macro jellyfish, include the order Rhizostomeae, comprising a clade of 91 species that contain more proteins than other scyphozoans [12,32,37]. Rhizostomes are generally characterized by the absence of marginal tentacles, in contrast to species of the order Semaeostomeae (Cnidaria: Scyphozoa) [38,39]. They also feature a central mouth opening and possess eight oral arms with numerous small apertures [32,40].

**Figure 2:**
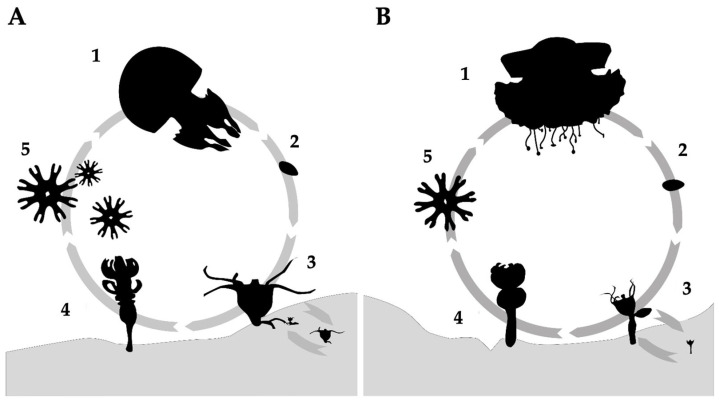
Life cycle of the order Rhizostomeae. (**A**) *Rhizostoma pulmo* exhibiting oligodisc strobilation; (**B**) *Cotylorhiza tuberculata* exhibiting monodisc strobilation. Life cycle stages: (1) mature medusa; (2) planula, (3) polyp, (4) polyp undergoing strobilation, and (5) ephyra.

Schiariti et al. (2024) [41] reviewed a total of 28 species from the order Rhizostomeae with described life cycles, representing approximately 30% of the currently recognized species. Most Rhizostomeae exhibit metagenetic life cycles, consisting of two distinct stages: a benthic phase and a free-swimming pelagic phase (Figure 2) [42,43]. Eggs and sperm from mature jellyfish, generally with noticeable sexual dimorphism (except for some exceptions) [41], are released into the water column to produce fully-grown planulae [43]. Fertilization can also occur inside the females for brooding species (internal fertilization) [44]. Typically, the initial settlement of planulae on the substrate occurs within 1–7 days, after which they metamorphose into scyphistomae, marking the beginning of the benthic stage [41].

Scyphistomae can propagate through various forms of asexual reproduction (e.g., lateral budding, podocyst formation, longitudinal fission, lateral budding by the stolon, motile bud-like tissue particles, and swimming buds) [41,43,44,45,46]. When environmental conditions are favorable, polyps fission perpendicularly in furrows, releasing ephyrae into the water column through the process of strobilation, thereby initiating the free-swimming phase [42]. Strobilation rates have been recently reevaluated according to the number of ephyrae per strobila and classified into four categories: myriadisc, polidisc, oligodisc, and monodisc [47,48]. In Scyphozoa, oligodisc strobilation (producing an average of 1–10 ephyrae) (Figure 2A) and monodisc strobilation (producing a single ephyra) (Figure 2B) are characteristic of the order Rhizostomeae [47,48].

### 3.2. Jellyfish Species

**Figure 3:**
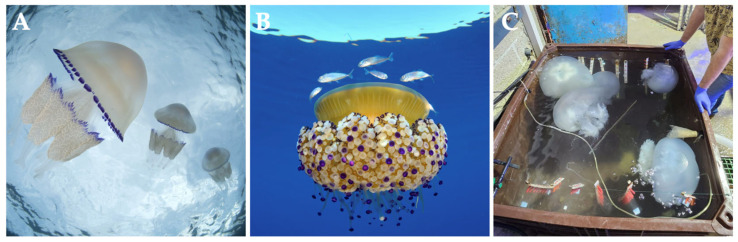
Rhizostomeae jellyfish species rich in collagen present in the Mediterranean Sea. (**A**) *Rhizostoma pulmo*; (**B**) *Cotylorhiza tuberculata*; and (**C**) *Rhopilema nomadica*. Photos (**A**,**B**) courtesy of David Antoja and (**C**) Dror Angel.

The Mediterranean Sea is recognized as a biodiversity hotspot, harboring a diverse mix of temperate and subtropical biota, along with a significant proportion of endemic species [49]. Among the Rhizostomeae jellyfish, the barrel jellyfish *Rhizostoma pulmo* (Figure 3A) is the most abundant species, found across both the Eastern and Western Mediterranean basins [50,51,52]. *R. pulmo* is the largest jellyfish species, with an umbrella diameter reaching up to 40 cm [53], responsible for forming blooms [43,51]. In its life cycle, planulae develop within the female gastrovascular system, and the polyp primarily propagates through podocysts, as well as by longitudinal fission, motile bud-like tissue particles, and lateral budding [43,45]. Each polyp can generate up to eight ephyrae by oligodisc strobilation (Figure 2A) [43,48]. Small jellyfish are visible during the spring, while larger adult individuals are more evident during the summer and early autumn along the Mediterranean coast [53].

Over the past decade, *R. pulmo* has exhibited marked and rapid changes in both phenology and population size, associated with rising water temperatures driven by global warming [45,52,54]. Experimental data show that higher temperatures result in a greater production of buds and ephyrae per *R. pulmo* medusa compared to lower temperatures [45,54]. *R. pulmo* has the highest collagen yield, particularly from the oral arms, ranging from 2.61 to 10.3 mg of collagen per gram of wet tissue. Both its abundance and high collagen content make it the most important species as a collagen resource in the Mediterranean Sea.

Similar to *R. pulmo*, *Cotylorhiza tuberculata* (Figure 3B) forms large blooms in the Mediterranean Sea [51,55]. Recent records have identified occurrences in the Marmara Sea [56] and along the Moroccan Northwest Mediterranean coast [57]. Adult *C. tuberculata* jellyfish, with an umbrella diameter reaching up to 35 cm, are more abundant in late summer and early autumn along the Mediterranean coast [53]. It is one of the few species that host zooxanthellae algae, which are present in all stages of its metagenetic life cycle, except for the planula stage [55]. *C. tuberculata* develops planulae inside the female gastrovascular system, and polyps reproduce asexually mainly by free-swimming buds [55]. Unlike *R. pulmo*, each polyp only produces a single ephyra by monodisc strobilation (Figure 2B). Although yielding less collagen than the barrel jellyfish, *C. tuberculata* also stands out for its high collagen content when compared with other scyphozoans, with a yield of 1.94 mg per gram of wet tissue in the oral arms [22].

Widely distributed in the Eastern Mediterranean, *Rhopilema nomadica* (Figure 3C), with an umbrella diameter reaching up to 80 cm [53], is a Lessepsian species recorded as NIS that has migrated from the Red Sea into the Mediterranean through the Suez Canal [58,59]. The westernmost record has been observed in the Western Mediterranean, near Sicily and Sardinia. Since its arrival in the basin, the population of *R. pulmo* has declined in the eastern basin [50,59]. Sexual reproduction, characterized by external fertilization, presumably occurs mostly during the summer swarming events in June and July [7]. During August and September, a new generation of polyps is formed [7]. Asexual reproduction occurs exclusively through podocysts, and, during strobilation, polyps produce up to six ephyrae, classifying them as exhibiting an oligodisc strobilation type [7,58,60]. Although it is part of the genus *Rhopilema*, which includes one of the most exploited jellyfish species in Asian countries (i.e., *Rhopilema esculentum*), known for its collagen content and quality [61], there are no studies that have specifically characterized *R. nomadica* collagen or evaluated collagen extraction yields for this species.

Another bloom-forming jellyfish found in the Mediterranean, although less abundant, is *Rhizostoma luteum* [44,57], which may have become increasingly frequent in certain Mediterranean areas over the past two decades [62]. Additionally, several NIS in the basin include *Phyllorhiza punctata* [63], *Catostylus tagi* [50], and the upside-down jellyfish *Cassiopea andromeda* [63], the latter of which has established itself in isolated locations such as Palermo Harbour (Italy), where small blooms now occur annually [9].

The collagen in *C. tagi* has been thoroughly studied, with its content estimated at 2.7% of dry weight [64], whereas the collagen in *R. luteum*, *C. andromeda*, and *P. punctata* has yet to be quantified and characterized using common techniques such as sodium dodecyl sulfate–polyacrylamide gel electrophoresis (SDS-PAGE) or amino acid composition. Further research is necessary to fully understand their yields and properties.

## 4. Jellyfish Collagen Extraction Methods

### 4.1. Preparation and Pre-Treatment

Jellyfish preparation begins with thorough cleaning and rinsing, usually with tap water and/or distilled water, to ensure the starting material is free of sand, debris, and contaminants [19,65]. Afterward, some authors separate the jellyfish into the umbrella and oral arms [66,67], while others use the entire organism [68]. Regardless of preparation, jellyfish can be used directly [69] or stored at −20 °C [65] or −80 °C [68] until needed for further processing. To our knowledge, no published studies have addressed the comparative effect of different storage conditions of jellyfish on the structural integrity and quality of extracted collagen, or on the extraction yield.

Since jellyfish are composed of approximately 97% water [70], their high water content significantly impacts collagen solubility [19]. To enhance solvent penetration and improve treatment efficiency during the extraction phase, processes such as size reduction [69,71], homogenization [72], or freeze-drying [22,73] can be applied to jellyfish tissues. These steps facilitate collagen extraction by increasing the rate of mass transfer within the tissue matrix, thereby improving collagen purity and enhancing the efficiency of subsequent chemical pre-treatment processes [74].

Chemical pre-treatment is typically required to remove non-collagenous proteins, pigments, and fats before collagen extraction [19]. However, jellyfish naturally contain lower amounts of these substances and have a less fibrous structure compared to mammalian and other marine sources, which is an advantage in the extraction process [21]. Because of this, some researchers choose to skip this step and proceed directly to collagen extraction [22,67,69,72,75], streamlining the process and enabling the development of more cost-effective and environmentally friendly methods. When pre-treatment is applied, the most commonly used solution is 0.1 M sodium hydroxide (NaOH) [65,66,76], which does not cause structural changes to the collagen chains [67,68,71]. Jellyfish can be pre-treated with NaOH through washes [66,73], incubated for several hours [65], overnight [77], or even 2 days [76]. As an alternative pre-treatment, Balikci et al. (2024) [71] dehydrated small umbrella fragments by immersing them in 99.9% ethanol overnight.

### 4.2. Extraction Process

The extraction process plays a crucial role in determining the yield, purity, and structural integrity of the final collagen product. To optimize these factors, various extraction methods have been developed, each with specific advantages depending on the desired application. Common methods include acid-based, enzymatic, and ultrasound-assisted extractions, which can be used alone or in combination for enhanced effectiveness, and each offering a set of advantages and disadvantages (Table 1).

#### 4.2.1. Acid-Based

The mechanism of collagen extraction under acidic conditions is mainly based on the increase in repulsive forces between tropocollagen molecules, leading to the solubilization of the less cross-linked collagen fractions [78]. This process facilitates the breaking of intramolecular and intermolecular non-covalent bonds by non-selective chemical hydrolysis, without significantly compromising the primary structure of collagen chains [79]. For jellyfish collagen extraction, the conventional method involves using 0.5 M acetic acid (CH_3_COOH) with continuous agitation at 4 °C for 72 h [22,66,67,68].

However, Barzkar et al. (2024) [68] reported that extending the extraction time from 24 to 48 h increased the yield by 17.4%, whereas further extension had no noticeable effect. A slightly higher concentration (i.e., 0.6 M CH_3_COOH) has also been employed in some studies [71,72], but concentrations below 0.5 M CH_3_COOH result in lower extraction yields [68]. In an effort to enhance the extraction efficiency, several researchers have proposed a two-stage extraction process [77,80]. Nevertheless, it has been demonstrated that the yield of acid-soluble collagen is more pronounced when this extraction method is integrated with other extraction techniques [66].

#### 4.2.2. Pepsin

Enzymatic extraction is an effective strategy for collagen recovery, as it improves solubility, optimizes extraction efficiency, and lowers antigenicity while maintaining the collagen’s structural integrity [81]. By selectively removing telopeptides, this method disrupts acid-resistant crosslinks in tropocollagen chains without altering the non-helical regions. Moreover, it contributes to the degradation of unwanted co-extracted proteins, leading to a higher-purity collagen extract [78].

Among the enzymatic methods, pepsin-assisted extraction is the preferred method for obtaining pepsin-soluble collagen from jellyfish. Pepsin-assisted extraction typically involves 0.5 M CH_3_COOH combined with pepsin at concentrations ranging from 0.05% to 10.0%. This method is commonly employed as a secondary step following an acetic-based method to solubilize residual collagen that remains insoluble under acidic conditions [69,72,73,76].

To prevent excessive collagen degradation, proper enzymatic inactivation is crucial and is typically achieved through dialysis against 0.02 M disodium hydrogen phosphate (Na_2_HPO_4_) for 3 days. While enzymatic extraction can improve yield and reduce processing time, it is more expensive than conventional acetic acid extraction, resulting in higher costs.

#### 4.2.3. Ultrasonic Assisted

Ultrasound-assisted extraction has been shown to be a highly effective technique for collagen recovery, improving mass transfer, tissue disruption, solvent penetration, and enzymatic activity in a wide range of marine sources [82]. In jellyfish collagen extraction, a combination of 15 min of sonication followed by one hour of mechanical mixing in acetic acid has been shown to effectively enhance cell disruption without compromising the triple helix structure of collagen [66]. This approach reduced the need for re-extractions, enhanced product purity, and produced up to seven times more collagen than acid-assisted extraction, and twice as much as the pepsin-assisted process [66].

Similarly, the application of ultrasonication to *R. pulmo* collagen extraction using 0.5 M CH_3_COOH and 1% pepsin led to a 47% dry weight yield, while significantly shortening the extraction time to just 3 h [65].

**Table 1:** Comparative summary of advantages and disadvantages of collagen extraction techniques applied to Rhizostomeae jellyfish found in the Mediterranean Sea.

Collagen Extraction Method		Advantages	Disadvantages	Jellyfish Species	References
Acid soluble collagen	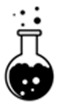	No damage to collagen structure Cost-efficient Simple process	Long extraction time Low yield Darked protein coloration	*Rhizostoma pulmo*, *Cotylorhiza tuberculata*, *Cassiopea andromeda*, *Catostylus tagi*	[22,66,79,83,84]
Pepsin soluble collagen	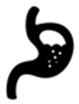	Increased yield Non-flamable Non-toxic	High cost Difficult to scale up	*Rhizostoma pulmo*, *Cassiopea andromeda*, *Catostylus tagi*	[79,83,84,85]
Ultrasonic assisted	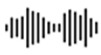	No damage to collagen structure Better purity Increased yield Reduced extraction time	Limited industrial scalability Complex steps	*Rhizostoma pulmo*	[65,66,79]

#### 4.2.4. Green Extraction Methods

New methods, such as supercritical fluid extraction (SFE) and deep eutectic solvents (DESs), are increasingly being employed to extract marine collagen, with the goal of minimizing environmental impact. SFE uses supercritical fluids like carbon dioxide (CO_2_) under high temperature and pressure to efficiently separate collagen from biological matrices. Compared to traditional methods, SFE offers several advantages, including non-toxicity, enhanced penetration, and higher yields, boosting collagen extraction from marine sponges by more than 30% compared to acid-based and acid/enzymatic methods [86,87].

On the other hand, DESs are biodegradable, low-toxicity mixtures of hydrogen bond donors and acceptors that remain liquid at room temperature due to their significantly lowered melting point [88]. This method has been used to extract collagen from cod skin, providing better yield, higher purity, and improved thermal stability [89]. As an innovative and environmentally friendly method, Batista et al. (2022) [90] extracted collagen from blue shark skins, reducing extraction time by 96 times and increasing yield by 2.5 times using natural deep eutectic solvents (NADESs) made from citric acid (C_6_H_8_O_7_) and xylitol (C_5_H_12_O_5_).

However, challenges remain with DESs, including high viscosity and mass transfer limitations [88], making optimization for large-scale applications still complex. Neither of these techniques has been explored for jellyfish collagen extraction, emphasizing the need for further research into sustainable alternatives.

### 4.3. Recovery, Purification, and Stabilization

After the extraction phase, collagen undergoes a series of recovery, purification, and stabilization steps. Salting out with 0.9 M sodium chloride (NaCl) causes collagen to precipitate from the solution. The resulting precipitate is collected by centrifugation and resuspended in 0.5 M CH_3_COOH.

Next, the collagen is dialyzed, either against 0.1 M CH_3_COOH [22], sequentially against 0.1 M, 0.05 M, and 0.025 M CH_3_COOH [65], or against 0.1 M CH_3_COOH followed by distilled water [71]. Finally, freeze-drying stabilizes the collagen, turning it into a powder for storage and later application.

## 5. Application of Jellyfish Collagen

### 5.1. Nutraceutical

Jellyfish consumption has documented healthy benefits [91]. Scientific studies have shown that edible jellyfish contain collagen that, when hydrolyzed, generates bioactive peptides with antioxidant and anti-inflammatory effects [92,93,94]. Type I collagen is primarily consumed to improve skin and bone health, whereas Type II collagen is commonly used to alleviate joint conditions such as arthritis [95]. For over a thousand years, jellyfish have been harvested in China for human consumption, and the country remains the global leader in both jellyfish production and consumption [96].

While jellyfish consumption is particularly popular in China, it has also spread to other Asian countries, leading to the expansion of edible jellyfish fisheries (e.g., *R. esculentum* and *Nemopilema nomurai*) in Southeast Asia since the 1970s due to growing demand from China, Japan, and Korea [37,96]. Due to their large, tough, and rigid bodies, the order Rhizostomeae is the main jellyfish consumed, which includes at least 29 of the 35 total edible jellyfish species [37,97]. They are favored for their distinctive crunchy and crispy texture, which is characteristic of comestible jellyfish products [98].

Jellyfish are rarely consumed as fresh ingredients, and they are typically processed within hours of being caught to prevent spoilage [98]. Processing typically involves weighing, washing with seawater to remove the mucus, sand, and potential bacteria, and removing the gonads, followed by soaking the jellyfish in large vats or tanks containing different mixtures of salt and alum [97,98,99,100]. The final semi-dried product can be stored for between 10 and 30 days, with a total weight reduction of up to 30% in the case of *Rhopilema hispidum* [99].

In the Mediterranean Sea, research has focused on identifying rhizostomeae species such as *R. pulmo* [92,93], *R. luteum* [94], and *C. tuberculata* [93] for their notable antioxidant activity and nutraceutical potential. This property is primarily attributed to the protein and phenol content, although other unidentified compounds may also contribute [93]. *R. luteum* demonstrated the highest antioxidant activity, which could be linked to its protein and phenol levels, showing an antioxidant activity of 2379 nmol of TE/mg of protein. Prieto et al. (2019) [94] highlighted that this value is twice that reported for *R. pulmo* and *C. tuberculata*, suggesting that the inherent protein properties of *R. luteum* play an important role in its high antioxidant activity.

A study of 1445 Italians analyzed their attitude toward jellyfish consumption in the Mediterranean Sea, considering factors such as age, gender, education, travel habits, and personality traits. Young people, frequent travelers, and those with higher education showed greater acceptance. In contrast, food neophobia and sensitivity to disgust hinder acceptance [101]. Although acceptance is not universal, Mediterranean jellyfish species could be used not only for the Asian market but also as a new regional food resource in Europe [101]. Despite the potential use of jellyfish as food being supported by European novel food regulations (Regulation 2015/2283) [91], health benefits [92,93,94], improvements in methods to process jellyfish [102], and the development of innovative culinary recipes [103], jellyfish remain absent from the traditional Mediterranean diet.

### 5.2. Cosmeceuticals

The cosmetics industry has grown significantly in recent years, offering a diverse range of innovative sources and active ingredients targeting specific customers and skin properties [104]. Collagen is one of the basic ingredients in the industry due to its humectant, moisturizing properties, and bioactivity [19,104]. The collagen peptides with a small molecular weight are capable of penetrating the skin [19].

In both the prevention of oxidative stress and protecting against UV radiation, jellyfish collagen stands out as a promising candidate for innovative products in the cosmetics industry [76,92,94]. Although research on jellyfish collagen for cosmetics is limited, the literature suggests that, with further study, jellyfish collagen and its derivatives could become valuable cosmetic ingredients [21].

### 5.3. Biomaterials

As the main structural protein in connective tissues, collagen plays a crucial role in tissue engineering and regenerative medicine by providing a natural scaffold for cellular organization and tissue repair [105]. Among alternative collagen sources, jellyfish-derived collagen has gained attention for its favorable biocompatibility [22,80,106], ability to promote cell proliferation [22,27,67], and potential to accelerate wound healing [107]. Its highly porous structure further facilitates the effective diffusion of nutrients and oxygen [80,108]. Often combined with crosslinking agents and bioceramics, it has been incorporated into advanced biomaterials designed for several biomedical uses (Table 2).

Among the jellyfish species inhabiting the Mediterranean region, *R. pulmo* collagen has been the subject of the most extensive research in the field of biomaterial development, exhibiting remarkable versatility across a range of biomedical applications. A significant proportion of this research has focused on medical-grade collagen from the company Jellagen^®^, Jellagen Cardiff, UK (Table 2). When combined with keratin and nano-hydroxyapatite, *R. pulmo* collagen forms an osteoinductive scaffold capable of sustaining mesenchymal stem cell viability and inducing spontaneous osteogenic differentiation in the absence of external induction agents [65]. The biocompatibility of *R. pulmo* collagen has also been validated in vivo, where collagen sponges exhibit effective tissue integration and elicit lower immune responses compared to bovine collagen. Notably, non-crosslinked forms demonstrate superior resorption, further highlighting their potential in biomedical applications [109].

On the other hand, *R. pulmo* collagen scaffolds have shown promise in oncological research, supporting ovarian cancer cell behavior and ECM marker expression at levels comparable to those observed with mammalian collagen-based alternatives [110]. In regenerative medicine, *R. pulmo* collagen scaffolds implanted in rats have been reported to enhance vascularization, elicit a reduced immune response relative to porcine collagen, and promote de novo bone formation [111]. Moreover, it has been explored in stem cell research, offering a stable, non-immunogenic microenvironment for microglial cells and demonstrating superior performance compared to rat tail collagen in terms of cell adhesion and viability [112]. Additionally, *R. pulmo* collagen also has demonstrated potential in cartilage regeneration, as it enhances chondroprogenitor cell proliferation and ECM deposition, with increased chondrogenesis in the presence of TGFβ1 [106]. Although genipin-crosslinked hydrogels have improved structural stability in chondrocyte culture, the inherent natural variability of jellyfish-derived collagen remains a significant challenge [113].

Although the collagen from *C. andromeda* has yet to be quantified, biomaterials from this jellyfish have been developed, including scaffolds and hydrogels (Table 2). Fernández-Cervantes et al. (2020) [114] elaborated decellularized scaffolds that preserve collagen structure and support fibroblast adhesion and proliferation, although further studies are needed to assess degradation and immunological response. Hydrogels combining *C. andromeda* collagen with synthetic polymers exhibit improved mechanical strength, blood compatibility, and antibacterial properties, while preserving the biocompatibility of jellyfish collagen, making them promising for regenerative medicine [84].

Despite the large blooms formed by *C. tuberculata* in the Mediterranean Sea [51], and a promising collagen yield [22], the development of biomaterial from this species has not yet been explored (Table 1), nor has it for *R. nomadica* and *P. punctata* (Table 2). Further research into their properties and biomedical applications could unlock new opportunities for sustainable and scalable collagen-based biomaterials, broadening the potential use of jellyfish in tissue regeneration and advancing regenerative medicine.

Jellyfish biomaterials, although limited when compared to vertebrate animals [115], have also been explored as effective carriers for drug delivery systems (Table 2). Charoenchokpanich et al. (2024) [116] produced hydrogels with jellyfish gelatin cross-linked with glutaraldehyde, which were more effective than those made with bovine and fish gelatin in the in vitro release of cephalozin, an antibiotic widely used as a prophylactic after surgical procedures. The authors did not report in vivo results.

For species inhabiting the Mediterranean Sea, Calejo et al. (2012) [83] used collagen from *C. tagi* to develop microparticles that were efficiently loaded with lysozyme and α-lactalbumin. Cross-linking with 1-ethyl-3-(3-dimethylaminopropyl) carbodiimide (EDC) resulted in the slow release of the entrapped proteins in in vitro assays.

**Table 2:** Collagen extraction methods, cross-linking strategies, and applications of various biomaterials derived from Rhizostomeae jellyfish found in the Mediterranean Sea.

Jellyfish Species		Collagen Extraction Method	Biomaterial Type	Additive	Cross-Linker	Biological Evaluation	Application	References
*Rhizostoma pulmo*	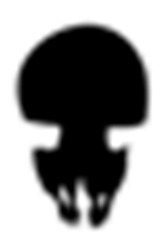	PSC	Scaffolds	Keratin/nano-spherical hydroxyapatite from eggshells	EDC/NHS and uncross linked	Human periodontal ligament fibroblast	Bone tissue	[85]
*Rhizostoma pulmo*	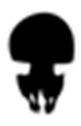	Jellagen^®^ *	Scaffolds	-	EDC and uncross linked	Wistar rats	Wound healing	[109]
*Rhizostoma pulmo*	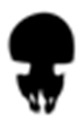	Jellagen^®^ *	Scaffolds	-	EDC	Ovarian cancer cells	Cell culture	[110]
*Rhizostoma pulmo*	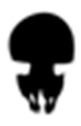	Jellagen^®^ *	Scaffolds	-	EDC/NHS	Wistar rats	Bone tissue	[111]
*Rhizostoma pulmo*	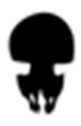	Jellagen^®^ *	Collagen solution	-	-	Human induced pluripotent stem cells (UKBi005-A)	Cell culture	[112]
*Rhizostoma pulmo*	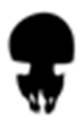	Jellagen^®^ *	Scaffolds	-	EDC	Bovine chondroprogenitor cell	Cartilage tissue	[106]
*Rhizostoma pulmo*	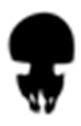	Jellagen^®^ *	Collagen solution and scaffolds	-	EDC	L929 fibroblasts and MC373-E1 pre-osteoblasts	Bone tissue	[117]
*Rhizostoma pulmo*	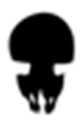	Jellagen^®^ *	Hydrogel	-	Genipin	Chondrocytes	Cell culture	[113]
*Rhizostoma pulmo*	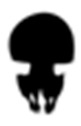	Jellagen^®^ *	Hydrogel	Chitosan/ fucoidan	-	Chondrocyte-like cells (ATDC5)	Cartilage tissue	[118]
*Rhizostoma pulmo*	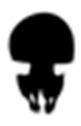	Jellagen^®^ *	Collagen solution	-	-	Fibroblast and pre-osteoblasts	Cell culture	[119]
*Rhizostoma pulmo*	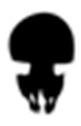	ASC	Scaffolds	Chitosan	-	Rat embryonic liver cells	Liver tissue	[77]
*Rhizostoma pulmo*	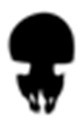	ASC	Hydrogel	-	HRP/H_2_O_2_	Nasal Chondrocytes and MC3T3-E1 pre-osteoblastic	Cartilage tissue	[120]
*Rhizostoma pulmo*	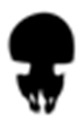	Jellagen^®^ *	Hydrogel	-	EDC/NHS; EDC/sNHS; PEG; Genipin	Immortalized human mesenchymal stem cells	Regenerative medicine	[121]
*Rhizostoma pulmo*	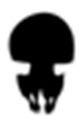	Jellagen^®^ *	Collagen solution	-	-	Primary fibroblasts, HT-1080 human fibrosarcoma line and Y201 mesenchymal stem cells	Cell culture	[27]
*Rhizostoma pulmo*	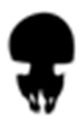	ASC, PSC	Collagen solution	-	-	Human fibroblasts and MG-63 osteosarcoma cells	Cell culture	[22]
*Cassiopea andromeda*	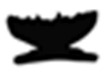	ASC, PSC	Scaffolds	TPU	-	Human monocytes and porcine dermal fibroblasts	Regenerative medicine	[84]
*Cassiopea andromeda*	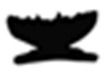	ASC, PSC	Hydrogel	TPU	-	Human monocytes and porcine dermal fibroblasts	Wound healing	[122]
*Cassiopea andromeda*	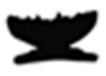	-	Scaffolds	-	-	Human fibroblasts	Skin tissue	[114]
*Catostylus tagi*	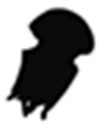	-	Hydrogel	-	EDC	-	Drug delivery	[116]
*Catostylus tagi*	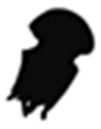	ASC, PSC	Microparticles	-	-	-	Drug delivery	[83]

Notes: PSC = Pepsin soluble collagen, ASC = Acid soluble collagen, EDC = 1-Ethyl-3-(3-dimethylaminopropyl) carbodiimide, NHS = N-hydroxysuccinimide, TPU = Thermoplastic polyurethane, sNHS = N-Hydroxysulfosuccinimide sodium salt, PEG = multi-arm functionalised polyethylene glycol (PEG)-derivatives, HRP = horseradish peroxidase, * Collagen purchased from Jellagen^®^, extraction method not specified.

## 6. Exploitation of the Resource

### 6.1. Active Fishing

At least 23 countries have been identified as involved in jellyfish fisheries, with 19 nations currently fishing for jellyfish. Worldwide landings of jellyfish reach at least 800,000 tons annually [98], exceeding those of many popular shellfish such as lobsters, clams, and mussels [123]. Although, in some cases, bottom trawlers are used [98,124], most fishing is performed on a small scale using artisanal vessels powered by outboard engines [96,99,100,125,126]. The activity is conducted nearshore, and the catch typically ranges from 1 to 5 tons of jellyfish [96,99,100,123,125,126]. In some areas, artisanal fishing is carried out with 1–3 trips per day, with crews of 2–8 fishermen [99,100,123].

Different fishing gears are used, including trawl nets, dip-nets, gillnets, set nets, drift nets, scoop nets, push-nets, purse-nets, beach-seines, weirs, and hooks [96,98,99,100,123]. Preserving the integrity of each catch is challenging due to the gelatinous, soft, and fragile nature of jellyfish [127], and their quality depends on the fishing method [128]. Buckets, gillnets, purse-nets, and dip-nets cause little or no damage to the catches, especially when compared to set nets [128]. Additionally, Jia et al. (2023) [127] found that bursiform-type anchor drift nets were superior for safely sampling large medusae of *Aurelia coerulea*, while plane-type anchor drift nets with a 100 mm mesh size proved more effective for capturing large medusae of the giant species *N. nomurai.* In terms of by-catch, dip-nets are highly selective, whereas set nets, purse seining, and, especially, trawling tend to generate larger amounts of non-target species [96].

Jellyfishery is characterized by large interannual fluctuations in abundance and biomass in the catch, and the fishing season is restricted to a few months, which vary by locality [96,99]. Depending on the species and the target market, the entire body, only the umbrella, or just the oral arms are processed [96,98,100,123,125]. For instance, along the Gujarat coast in India, fishers focus primarily on the oral arms and throw the bells back into the sea [99]. In Southeast Asia, fishermen bring the entire bodies of Cilacap and Ball types to local processing factories, while, for the White type, only the umbrellas are loaded onto the boat after the oral arms are cut off at sea [125].

Unlike the Asian continent, jellyfish fishing is not a common or widely established practice in Europe. Between 1984 and 2006, Turkey operated the only large-scale commercial jellyfish fishery, capturing *Aurelia* spp., *R. pulmo*, and *R. nomadica* mainly for human consumption, with a contribution of 11% from the Mediterranean [98,128]. In 2014, commercial harvesting of the barrel *Rhizostoma octopus* began for the production of medical-grade collagen by Jellagen^®^ in Carmarthen Bay (United Kingdom) [129]. By 2015, the total catch reached 4.3 tons [129]. Today, the company appears to rely exclusively on *R. pulmo* for this purpose. While no data on total catches of this species have been found, the raw material seems to originate not only from the United Kingdom but also from the United States [130].

Norway considered harvesting *Periphylla periphylla*, but, despite its abundance in fjords, the lack of demand and market has prevented the development of a commercial fishery [128]. In 2006, Spain received a visit from a Japanese business group to evaluate the possibilities of exporting *R. pulmo*, *C. tuberculata*, and *Pelagia noctiluca* for food purposes to the Asian market. The proposal did not go forward due to the instability of exports caused by the spatio-temporal variations in the life cycles (J. Tena, personal communication). Also, in 2018, Israel conducted a pilot trial exporting ~100 kg of *R. nomadica* to China. However, a lack of investment and limited interest from regulatory bodies have stalled its commercial exploitation [128].

The overexploitation of jellyfish is a significant concern, as evidenced by historical declines, such as that of *R. esculentum* in China during the 1980s, likely driven by the extraction of the entire stock, and by the ongoing illegal and out-of-season harvesting that occurs annually in Liaodong Bay [131].

Towards the establishment of a European jellyfish fishery, including the Mediterranean Sea, Edelist et al. (2021) [128] highlight several critical considerations: (1) alignment with Sustainable Development Goal 14, (2) responsible management practices to ensure long-term sustainability, employing tailored strategies for both native and invasive species to maintain ecosystem stability, (3) adopting ecosystem-based fishery management under the ecosystem approach as a comprehensive framework for sustainable fisheries with a solid knowledge base on the demography, reproduction biology, and ecology of the fished jellyfish species and the ecosystem they inhabit, and (4) addressing socioeconomic factors, such as fair employment, safe working conditions for fishers and sufficient profit margins.

### 6.2. By-Catch

The frequency and severity of negative interactions between jellyfish blooms and the fishing industry appear to be increasing [5,11,13,14,132], with the North Pacific, followed by the Mediterranean Sea, being the most affected areas due to the giant *N. nomurai* and *Aurelia* spp. and *R. nomadica*, respectively [13].

Net clogging, sometimes resulting in breakages, catch deterioration, and the increase in fishing time, fuel consumption, and stings stand out among the most frequently reported direct impacts affecting fishing fleets worldwide [13,132,133,134]. Some jellyfish-exclusion devices used with fishing gear have been tested in several countries around the world, but with limited success [5]. Therefore, greater research efforts are needed to develop more effective technologies [133]. Although the impact of jellyfish blooms on fishing operations has been reported several times, information regarding their economic consequences remains limited [13].

**Figure 4:**
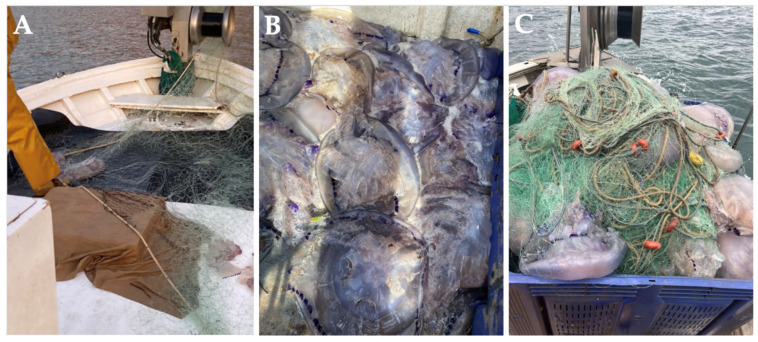
*Rhizostoma pulmo* jellyfish by-catches from Spanish artisanal fishermen of the COLMED project. (**A**) Pieces of jellyfish torn by fishing nets; (**B**) recovered jellyfish by-catches; and (**C**) fishing nets entangled with jellyfish.

Due to the undeniable value of jellyfish in various industries [93,135], D’Ambra and Merquiol (2022) [135] suggested promoting the valorization of jellyfish by-catch as a key strategy for their sustainable use within the framework of a circular economy and zero-waste policies. In the Mediterranean, researchers from the COLMED project are working closely with Spanish artisanal fishermen’s guilds to extract collagen from *R. pulmo* and *C. tuberculta* by-catches (Figure 4A–C) [136].

One of the main challenges they face is optimizing the use of this resource, as fishermen, when unintentionally capturing large quantities of jellyfish, often allow them to decompose for several days, using this as an effective method to untangle and clean the nets (A. Ballesteros, personal communication) (Figure 4C). Therefore, as pointed out by D’Ambra and Merquiol (2022) [135], effective management will be crucial to fully harness the potential of jellyfish by-catch in cooperation between scientists and fishermen.

*Rhizostoma pulmo* collagen, obtained as a by-catch from commercial fishing, has been incorporated into biomimetic membranes for liver tissue engineering. Its combination with methacrylated chitosan enhanced hepatocyte function and differentiation, further emphasizing the potential of marine discards as valuable sources of biomaterials (Table 2) [77].

### 6.3. Aquaculture

Jellyfish availability in the marine environment is unpredictable, and their distribution experiences considerable spatio-temporal variations [51,137]. Although jellyfish biomass seems to sustain the market demand over time in active fishing countries [135], the controlled production of jellyfish presents a promising alternative to ensure a continuous supply of biomass, free from unknown contaminants, with guaranteed traceability and sustainable exploitation [138,139,140,141].

Commercial jellyfish aquaculture systems for the cultivation of *R. esculentum* have been under development in China since the 1980s, achieving great success in the 1990s [61]. In 1984, hatchery-produced ephyrae were released into coastal waters to boost fishery yields, fo llowing the overexploitation of the species [142]. Due to its economic importance in China, *R. esculentum* is cultivated in ponds larger than 2 hectares in size and deeper than 1.5 m [61]. Its cultivation techniques are well established [61,143], allowing jellyfish to reach an umbrella diameter of up to 30 cm in 70 days [61]. In the Mediterranean Sea, a region far removed from the successful case of *R. esculentum*, full-scale jellyfish exploitation is still in its early stages, primarily due to the limited market demand for jellyfish-derived products. Currently, a few companies are distributing jellyfish for ornamental purposes [144].

In jellyfish farming, polyps are considered an inexhaustible source of jellyfish [139], as they reproduce asexually through the strobilation process (Figure 2) [42,43]. For this reason, and to maintain a continued stock, healthy polyp colonies are essential [139]. Polyp colonies can be obtained in various ways. The most common method is to collect adult medusae from the marine environment and encourage their natural spawning in a closed system [43,138,139], although planulae can also be collected directly from female medusae in brooding species [44] or through in vitro fertilization techniques [138,139]. Additionally, polyps can be purchased from jellyfish aquaculture companies [144] or provided by aquariums. The strobilation process occurs naturally but can also be induced [138,139].

Maintaining polyp colonies is relatively simple. The primary food consists of rotifers (*Brachionus* sp.) and/or newly hatched *Artemia* sp. nauplii, provided once a day [138,139,145] (Table 3). Along with regular weekly water changes in closed systems, this ensures the health of the colony and supports its asexual reproduction [138,139,145]. Polyps can also be kept in a flow-through system without the need for water changes in tanks [145] or in closed systems with water renewal [138,139].

The medusa phase of the life cycle requires more specific techniques than polyps, and it is considered more time-consuming by the staff [145]. Ephyrae are grown in glass flasks, plastic jugs, or petri dishes [44], but, from a mass production point of view, they should be cultivated in larger volumes of water using air-kreisels or kreisels in a closed or flow-through system [139,140,145]. Rotifers, with a smaller size than *Artemia* sp. nauplii. [138], are a suitable food for the first weeks [138,140,146], although some ephyrae are fed directly with newly hatched *Artemia* sp. nauplii (Table 3) [60,138,139].

In juvenile and adult stages, a varied diet is recommended for proper growth. Mysis, red plankton, bloodworms, mixed fish, fish eggs, and/or fish larvae can be added [138,139,147,148]. Moon jellyfish *Aurelia* spp. or the upside-down jellyfish *Cassiopea* spp. were also introduced into the diet of medusivorous jellyfish [138,139], although it has been shown that it is not necessary to close their life cycle in captivity [140]. Types of tanks vary between species, but the best-known for cultivating jellyfish is the kreisel type [138,139].

**Figure 5:**
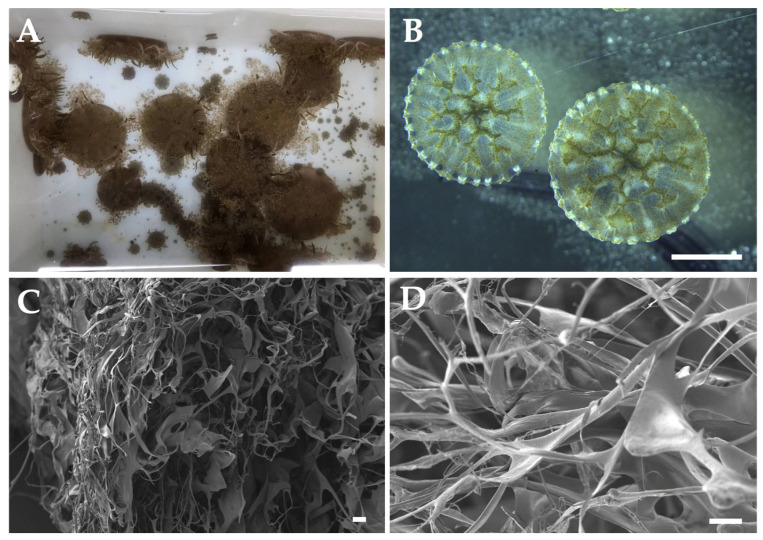
*Cassiopea* spp. scaffolds from farmed jellyfish. (**A**) *Cassiopea* spp. jellyfish; (**B**) ephyrae from polyps; and (**C**,**D**) Scanning Electron Microscope (SEM) scaffolds images. Scale bars: (**B**) 2 mm, (**C**) 20 μm, (**D**) 10 μm.

According to De Domenico et al. (2025) [141], *Cassiopea* spp. is both a desirable and potentially viable example of sustainable aquaculture, thanks to its ease of cultivation, high reproductive success, and the diverse range of chemical species with biological activity and potential applications (Figure 5A,B). De Domenico et al. (2025) [141] compared the biochemical composition of *C. andromeda* reared in an aquarium and wild jellyfish from Palermo (Italy). While soluble proteins showed no significant differences, total protein content, including collagen, varied after enzymatic digestion with pepsin and collagenase. Pepsin-digestible proteins showed the most variability, and wild jellyfish exhibited more peptides, including high-molecular-weight peptides (>100 kDa) and a band around 17 kDa. Additionally, to our knowledge, the first biomaterials derived from farmed jellyfish have been developed using *Cassiopea* spp. [149] (Figure 5C,D), enhancing the use of aquaculture jellyfish in blue biotechnology.

**Table 3:** Strobilation type and culture conditions Rhizostomeae jellyfish present in the Mediterranean Sea, including temperature, salinity, rearing tanks, and feeding regimen for each life cycle stage.

Jellyfish Species		Strobilation Type	T (°C)	Salinity	Tank				Foods Regimen				References
					Polyp	Ephyra	Juvenile	Adult	Polyp	Ephyra	Juvenile	Adult	
*Cassiopea* spp. *	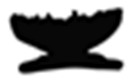	Monodisc	18–29	36–37, tolerant	WAK	WAK, MGT	MRT	MRT	R, AN	R, AN	R, AN	AN, WP	[45,48,138,139,145,148,150]
*Catostylus* spp.	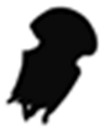	Oligodisc	19–28	32–37, tolerant	-	MGT	K, P-K	K, P-K, MRT	MG, R, AN	MM, R, AN	AN	AN, WP, FC	[48,139,151,152]
*Cotylorhiza* *tuberculata* *	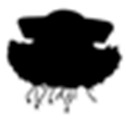	Monodisc	22–28	36–38, tolerant	WAK	WAK, MGT	P-K, MRT, RST	CT	R	R	AN	AN	[48,138,139,145]
*Phyllorhiza punctata* *	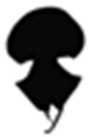	Monodisc	13–25	36–37	WAK	WAK	P-K, MRT, RST	K, P-K, CT	R, AN	R	AN	AN	[42,45,138,139,145,146]
*Rhizostoma* *luteum*	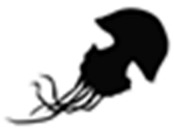	Monodisc	17–26	34–38	-	MGT	K	K	R	AN	AN	AN	[44,146]
*Rhizostoma pulmo*	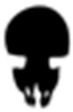	Oligodisc	13–28	36–37	WAK	WAK	K, P-K	MRT	R, AN	R, AN	AN, WP	AN, WP	[45,48,138,139,145]
*Rhopilema* *nomadica*	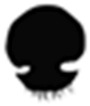	Oligodisc ^a^	21–29	39–40	-	K	K	K	AN	AN	AN	AN	[58,60]

Notes: T = Tank-Modified grow-out tank for ephyrae (MGT), Kreisel (K), Pseudo-Kreisel (P-K), Modified rectangular tank (MRT), Rectangular screened-in flow-through tank (RST), Cylindrical tank (CT), Water–air kreisel (WAK). Food regimen: Rotifers (R), *Artemia* spp. nauplii (AN), Wild plankton (WP), Frozen crustaceans (FC), Macerated femele mussel gondals (MG), Mashed mussel (MM). ^a^ Classified as oligodisc based on the production of from 5 to 6 ephyrae per polyp in Lotan et al. (1992) [58]. * Species with zooxanthellae. IED strip with photosynthetic spectrum (4500 K and 80 W). All life cycle stages require light.

## 7. Conclusions

Jellyfish collagen is an emerging and sustainable alternative to mammalian-derived collagen, offering exceptional biocompatibility, low allergenic risk, and significant potential for various industrial applications. In the Mediterranean Sea, jellyfish species such as *Rhizostoma pulmo*, *Cotylorhiza tuberculata*, and *Rhopilema nomadica* are gaining increasing attention not only due to their large blooms but also because they represent a valuable opportunity to harness these organisms.

Research on jellyfish species in the basin has primarily focused on the barrel jellyfish *R. pulmo*, with a strong emphasis on its nutraceutical benefits and promising applications as biomaterials in tissue engineering and regenerative medicine. However, jellyfish species like *C. tuberculata* and *R. nomadica*, which are known for their blooms in the Mediterranean, remain unexploited despite their significant potential for collagen-based products.

With the Mediterranean Sea being one of the regions most affected by the impact of jellyfish on the fishing industry, ongoing projects are being implemented in collaboration with fishermen’s guilds to promote circular and blue economy strategies, aiming to valorize jellyfish by-catch as an innovative resource. While jellyfish fishing is not as well-established as in Asian countries and faces challenges such as fluctuating populations and limited market demand, jellyfish aquaculture offers a promising alternative to ensure a continuous and sustainable supply with the Rhizostomeae *Cassiopea* spp. in the spotlight.

## Data Availability

Data sharing is not applicable. No new data were created or analyzed in this study.

## Abbreviations

ECM	Extracellular matrix
SFE	Supercritical Fluid Extraction
DES	Deep Eutectic Solvents
NADESs	Natural Deep Eutectic Solvents
NIS	Non-indigenous species
